# A Rare Case of Concurrent Urinothorax and Myelomatous Pleural Effusion

**DOI:** 10.1155/crpu/6880861

**Published:** 2026-03-11

**Authors:** Prachi Bhanvadia, Aarish Lalani, Sara Samadani, Amandeep Bawa, Laxman Wagle

**Affiliations:** ^1^ Department of Internal Medicine, Ascension Saint Agnes Hospital, Baltimore, Maryland, USA; ^2^ Department of Pulmonary Medicine, Ascension Saint Agnes Hospital, Baltimore, Maryland, USA

**Keywords:** CD138 positive plasma cells, extramedullary plasmacytoma, hemorrhagic pleural effusion, multiple myeloma, myelomatous pleural effusion, obstructive uropathy, pleural fluid analysis, urinothorax

## Abstract

**Introduction:**

Myelomatous pleural effusion (MPE) is a rare complication of multiple myeloma, occurring in < 1% of cases and associated with poor prognosis. Urinothorax, another rare cause of pleural effusion, results from urine leakage into the pleural cavity, typically due to obstructive uropathy or trauma. Although both conditions are individually uncommon, their concurrent occurrence has not been previously reported.

**Case Presentation:**

An 81‐year‐old man with relapsed multiple myeloma presented with dyspnea, hematuria, and acute renal failure. Imaging revealed bilateral hydronephrosis, obstructive uropathy from a pelvic mass, and bilateral pleural effusions. Thoracentesis showed an exudative, hemorrhagic effusion with a pleural fluid‐to‐serum creatinine ratio > 1, initially suggesting urinothorax. Despite bilateral nephrostomy, the effusion recurred. Cytology and immunohistochemistry confirmed MPE. Biopsy of the pelvic mass revealed extramedullary myeloma (plasmacytoma), which explained both the ureteral obstruction and pleural involvement.

**Conclusion:**

This is the first reported case of simultaneous MPE and urinothorax. It underscores the importance of a comprehensive, multidisciplinary diagnostic approach when evaluating complex pleural effusions with overlapping features.

## 1. Introduction

Pleural effusions are a common clinical finding with diverse etiologies, ranging from infectious and inflammatory processes to malignancies and systemic disorders. However, certain types of pleural effusions, such as myelomatous pleural effusion (MPE) and urinothorax, are exceedingly rare, making their concurrent occurrence an exceptional diagnostic challenge.

MPE is a rare complication of multiple myeloma (MM), occurring in less than 1% of cases due to the infiltration of malignant plasma cells into the pleural space. It typically presents as an exudative effusion and is associated with a poor prognosis, requiring cytological confirmation for definitive diagnosis [1].

Urinothorax, on the other hand, is a rare cause of pleural effusion characterized by the presence of urine in the pleural cavity, usually secondary to obstructive uropathy, trauma, or iatrogenic injury. It is often identified by a pleural fluid‐to‐serum creatinine ratio greater than 1 [2].

Despite their distinct pathophysiological mechanisms, both MPE and urinothorax share overlapping clinical and laboratory features, complicating the diagnostic process. The presence of hemorrhagic pleural fluid, renal impairment, and metabolic disturbances can further obscure the underlying etiology. Given that MPE is associated with a median survival of fewer than 4 months, prompt and accurate differentiation from other causes of pleural effusion is crucial for optimizing patient management [3].

The concurrent occurrence of MPE and urinothorax has not been previously documented in the medical literature, and its impact on clinical outcomes remains unknown. This case report is aimed at highlighting the importance of a systematic diagnostic approach in cases of complex pleural effusions and emphasizing the need for further research on the prognostic implications of such rare dual presentations.

## 2. Case

An 81‐year‐old male with a history of standard‐risk immunoglobulin G (IgG) lambda MM, previously treated with daratumumab and lenalidomide, presented to the hospital due to worsening dyspnea on exertion, occasional hematuria, and decreased urine output. Despite ongoing treatment, he had not achieved remission. On physical examination, the patient exhibited reduced breath sounds over the bilateral lower lobes, tenderness upon palpation over the bilateral flanks, and frank hematuria.

Upon admission, his complete blood count (CBC) was largely unremarkable, but his basic metabolic panel (BMP) showed significant abnormalities, including a potassium level of 7.0 mmol/L, creatinine level of 12.7 mg/dL, blood urea nitrogen (BUN) of 124 mg/dL, and bicarbonate (HCO3) of 12 mEq/L.

**Figure 1:**
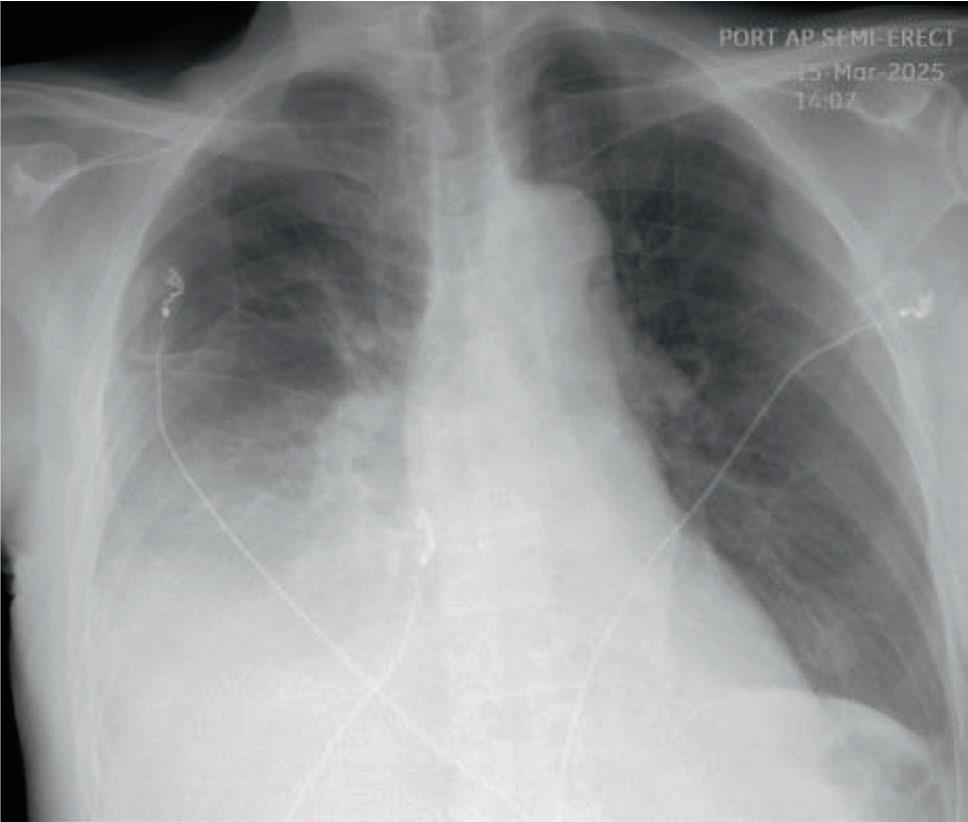
CXR on admission (3/15/2025).

As shown in Figure 1, chest X‐ray (CXR) revealed consolidation or pleural effusion in the right lower lobe.

**Figure 2:**
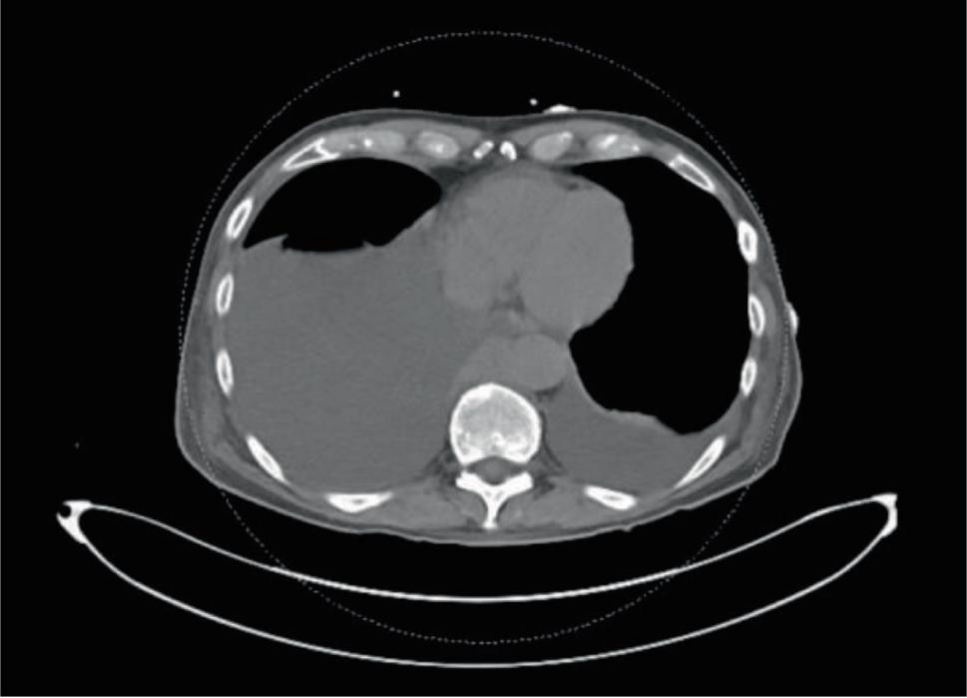
Showing moderate right and small left pleural effusion.

**Figure 3:**
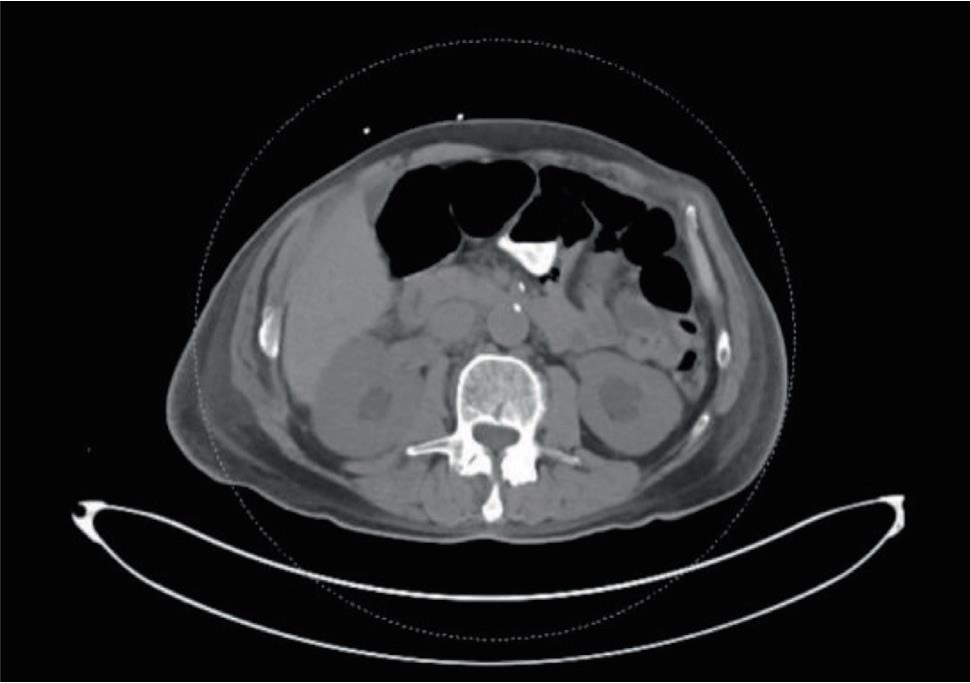
CT scan on admission showing bilateral hydronephrosis.

**Figure 4:**
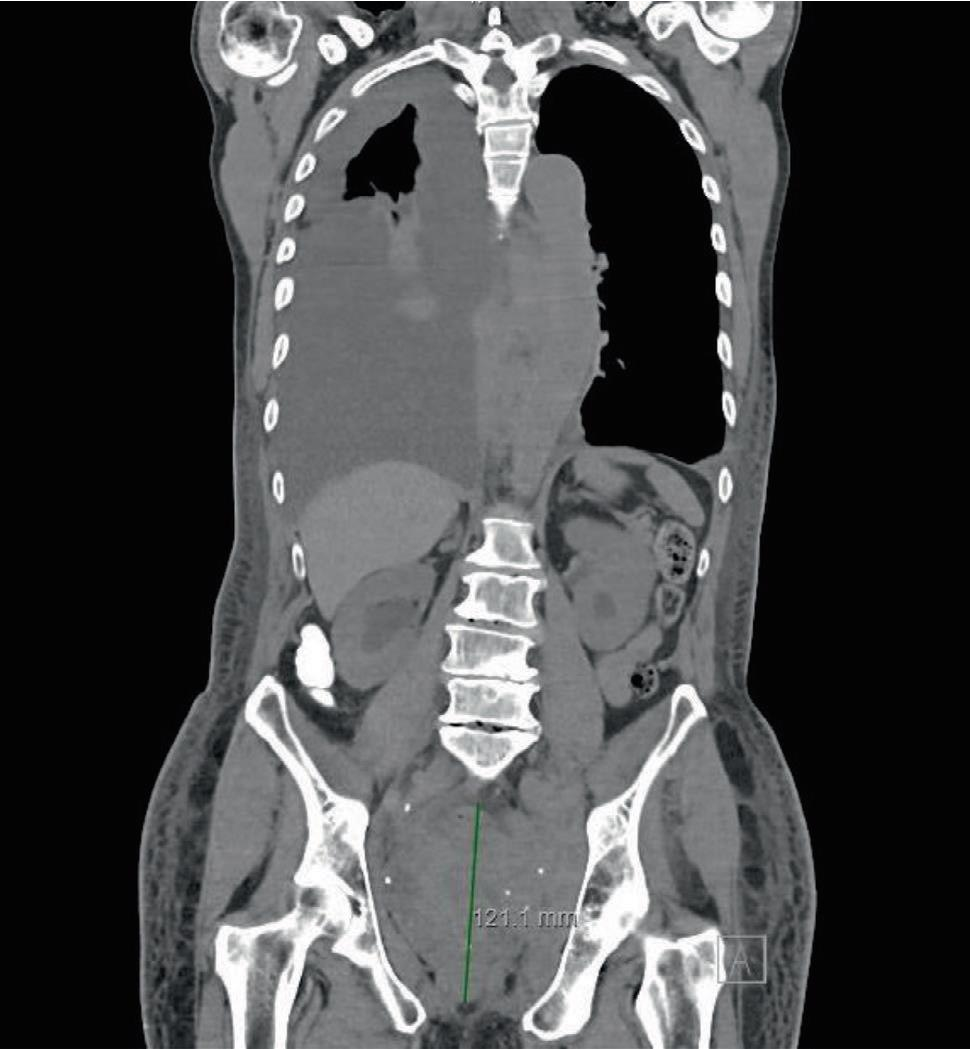
Soft tissue mass in the pelvic region.

**Figure 5:**
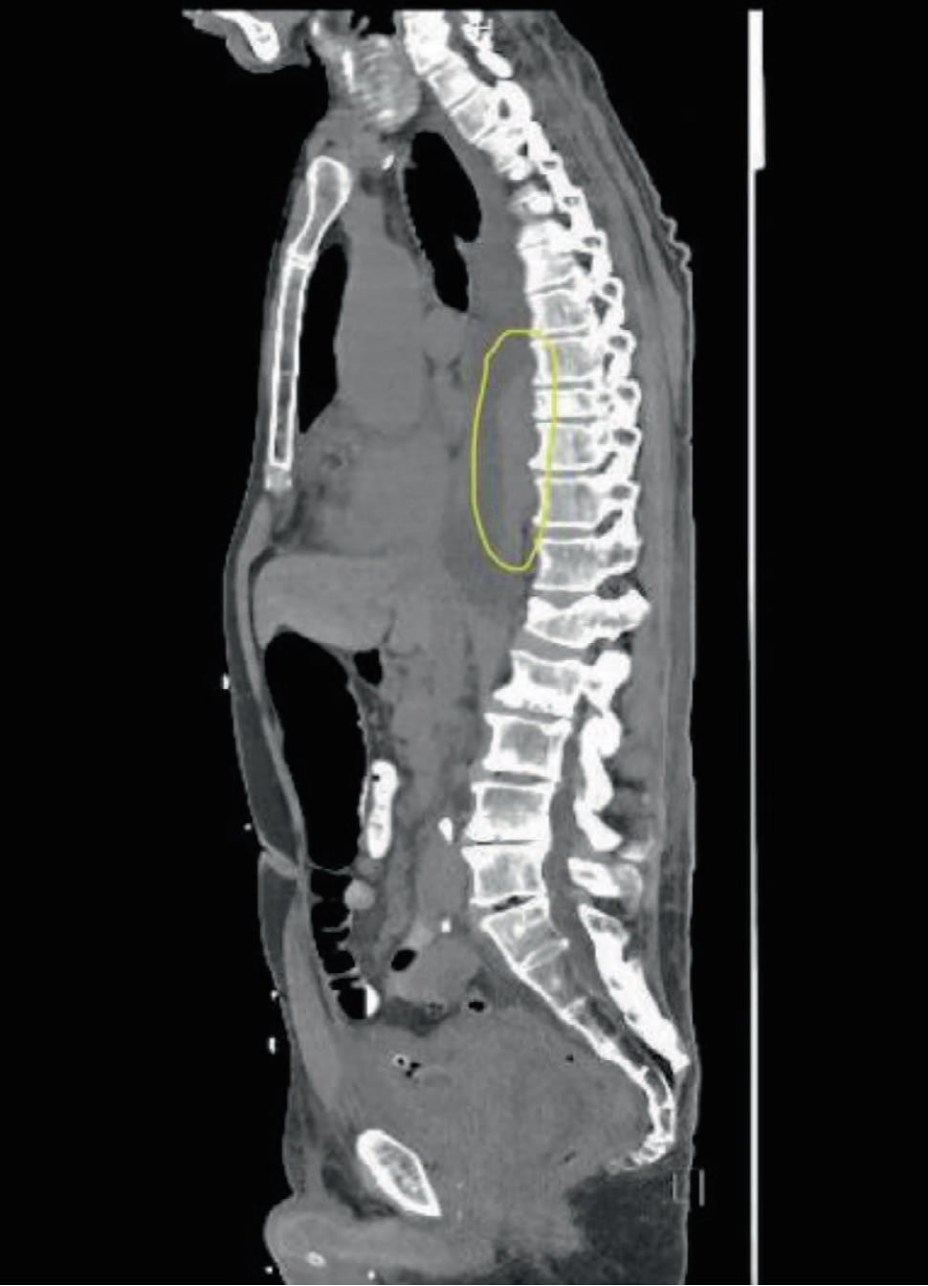
Mass anterior to T9.

Computed tomography (CT) of the chest, abdomen, and pelvis demonstrated moderate right‐sided pleural effusion, a small left‐sided pleural effusion (Figure 2), bilateral hydronephrosis and hydroureter (Figure 3), and diffuse thickening of the urinary bladder wall. Additionally, a soft tissue mass at the T9 vertebral body extending 1.9 cm beyond the bone was observed, and a lobulated soft tissue mass anterior to lumbar spine in size measuring 14.3 × 2.4 × 5.2 cm (Figures 4 and 5).

**Figure 6:**
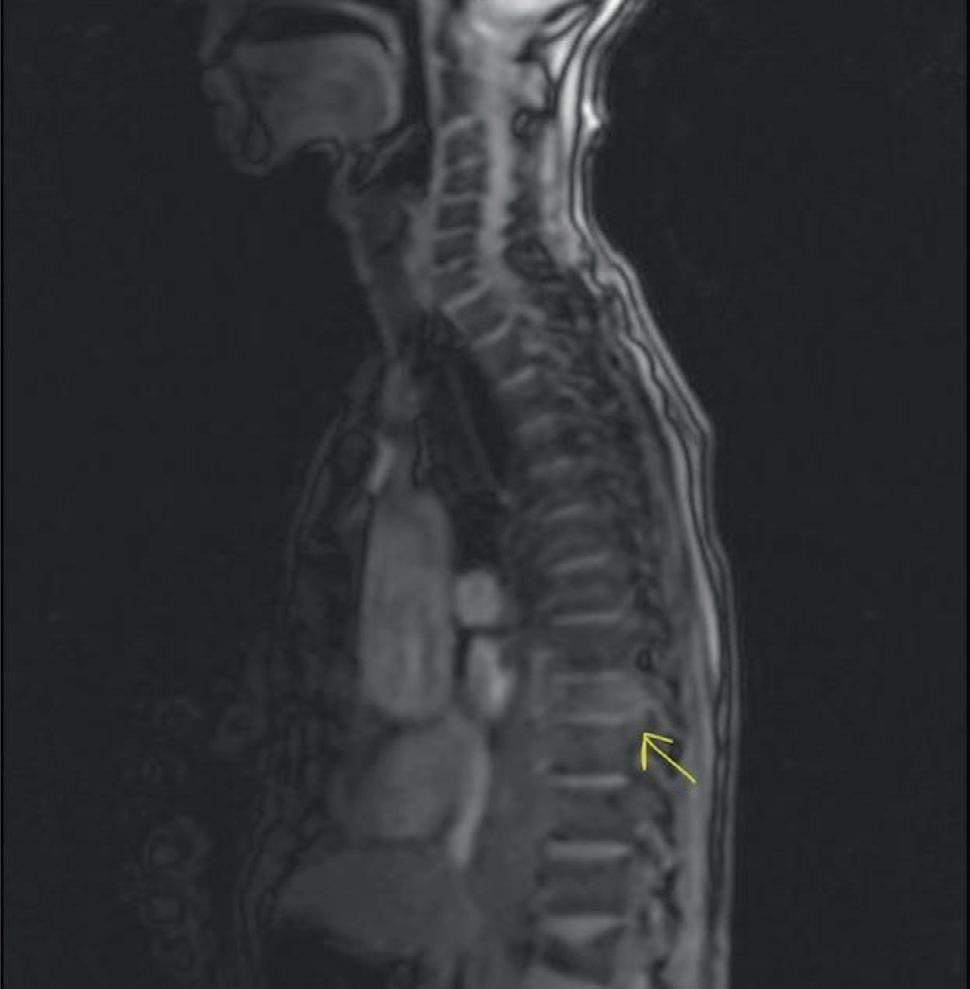
MRI showing the prevertebral mass over T9.

**Figure 7:**
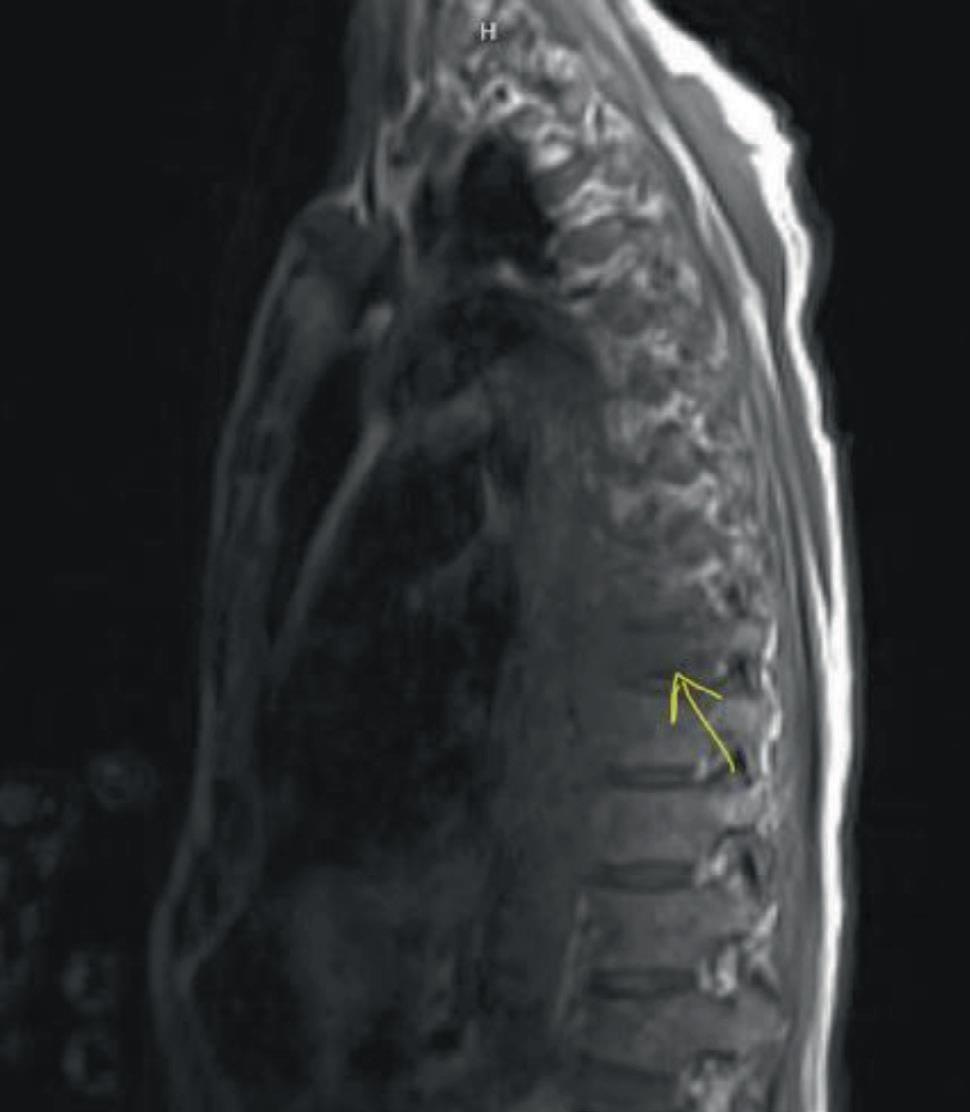
MRI showing the prevertebral mass over T9.

The enlarging prevertebral mass attributable to plasmacytoma with increase in size as compared with prior suggested disease progression. The same was confirmed by thoracic MRI as in Figures 6 and 7.

**Figure 8:**
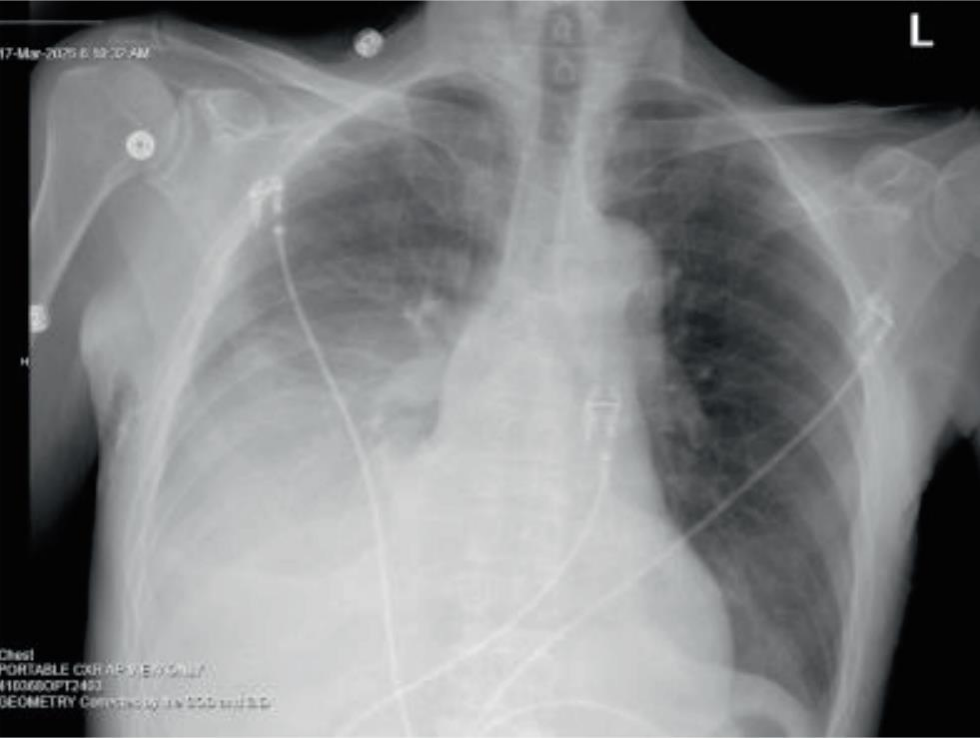
CXR post dialysis session 1.

**Figure 9:**
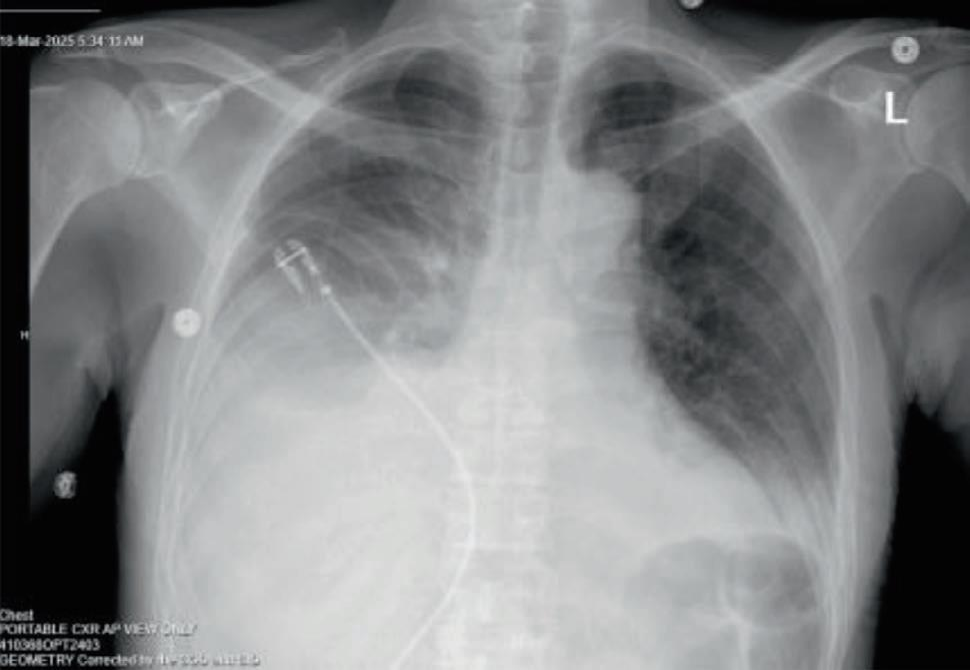
CXR post dialysis session 2.

**Figure 10:**
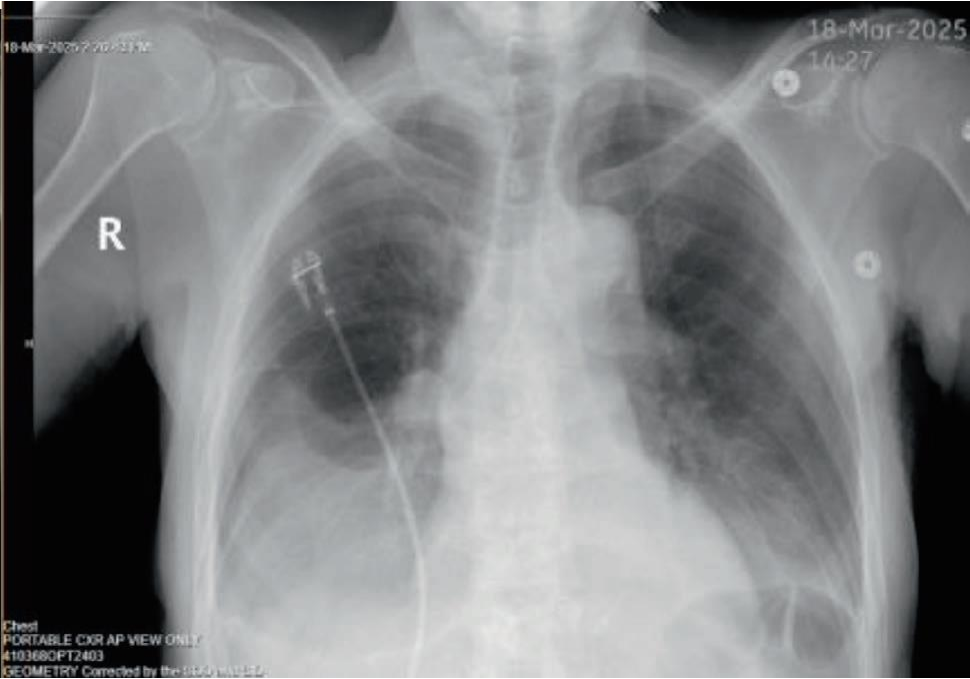
CXR post thoracentesis.

The patient′s worsening dyspnea was initially attributed to pleural effusion and volume overload from acute renal failure secondary to obstructive uropathy. So he was started on urgent dialysis. The decision for dialysis over nephrostomy tube placement at that time was made purely based on technical factors of easy availability of hemodialysis as it was a weekend. A urology consultation was requested for obstructive uropathy and nephrostomy tube placement. After three consecutive dialysis sessions, his creatinine level improved to 5.9 mg/dL, and his BUN decreased to 31 mg/dL. However, despite these interventions, his pleural effusion continued to worsen as shown in Figures 8, 9, and 10.

Given the persistence of the pleural effusion, a pulmonologist was consulted, and the decision was made to perform a thoracentesis.

During the thoracentesis, approximately 1.5 liters of hemorrhagic pleural fluid were drained and sent for analysis. On the same day, the patient also underwent placement of bilateral nephrostomy tubes, which produced hemorrhagic output similar in appearance to the patient′s hematuria.

**Figure 11:**
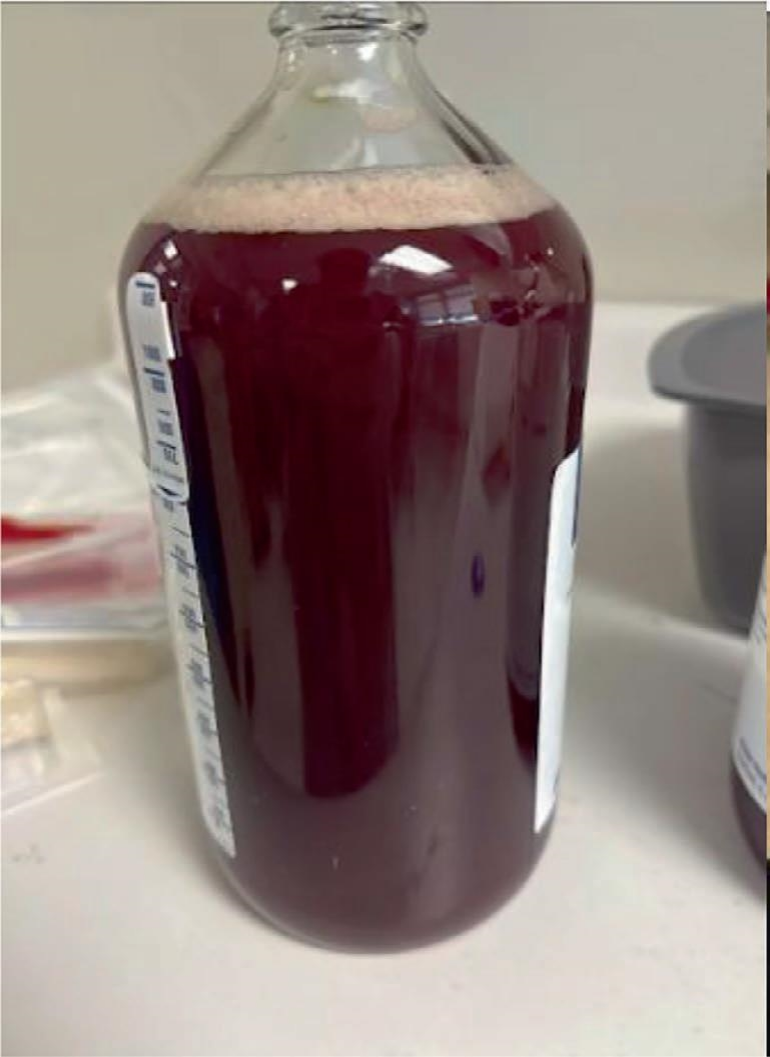
Sample from thoracentesis.

**Figure 12:**
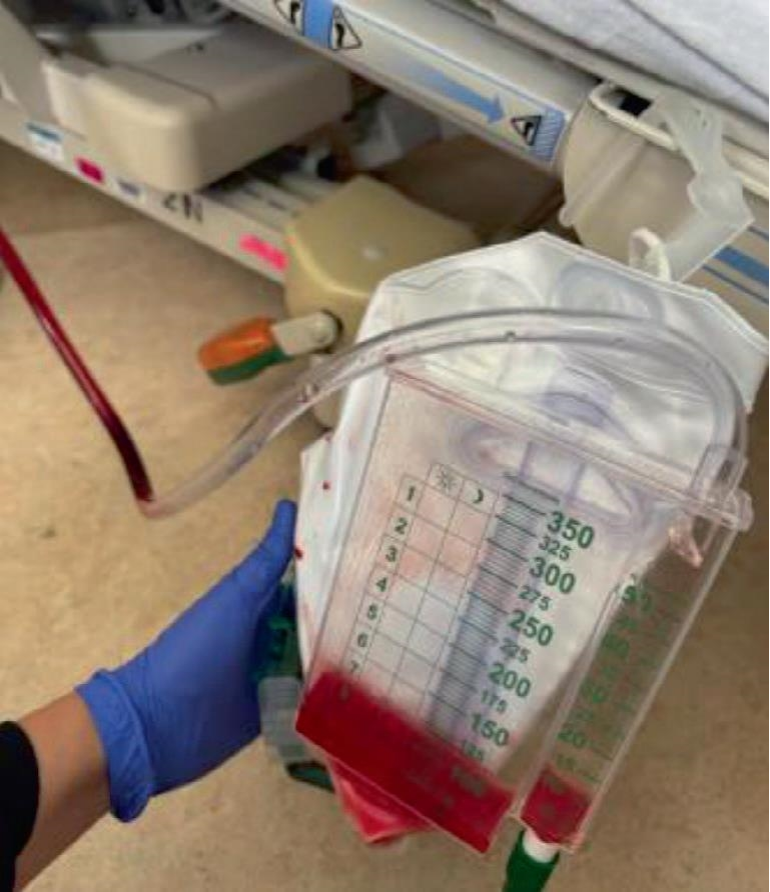
Urine output.

**Figure 13:**
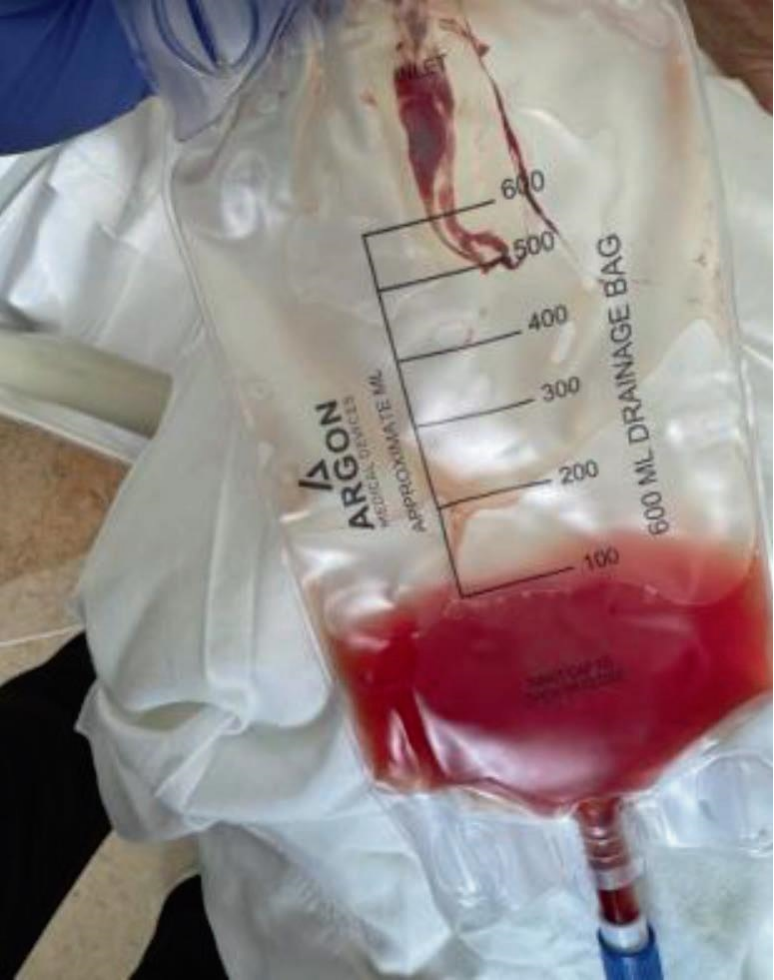
Left nephrostomy output.

**Figure 14:**
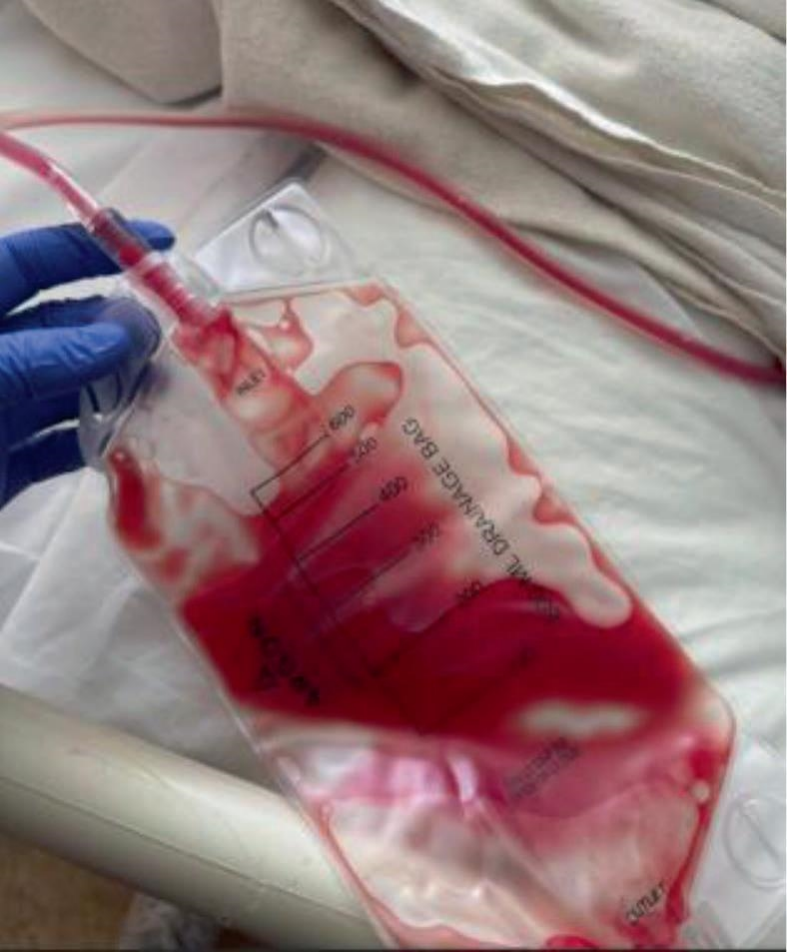
Right nephrostomy output.

Figure 11 (sample from thoracentesis), Figure 12 (urine output), Figures 13 and 14 (nephrostomy output) shows the similarity in the appearance of the fluid from the pleural cavity and urine, raising the suspicion of urinothorax.

Pleural fluid analysis revealed exudative characteristics, with a lactate dehydrogenase (LDH) level of 1766 U/L, total protein of 4.6 g/dL, glucose level of 48 mg/dL, red blood cell (RBC) count of 58,000 cells/*μ*L, and white blood cell (WBC) count of 395 cells/*μ*L with 96% mononuclear cells. Pleural fluid creatinine was 5.9 mg/dL. Serum creatinine around the same time was 5.9 mg/dL, which subsequently improved to 4.4 mg/dL the following day. Given that the pleural fluid‐to‐serum creatinine ratio was > 1, the pleural effusion was initially considered to be a possible urinothorax, secondary to obstructive uropathy.

**Figure 15:**
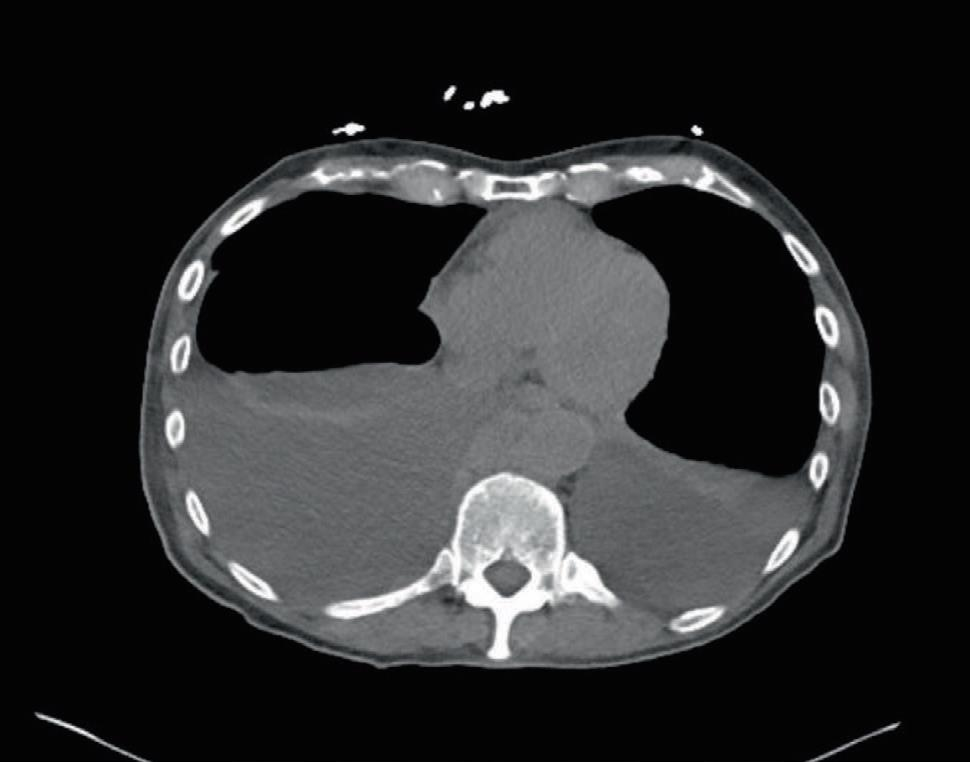
Showing recurrent pleural effusion (3/20/2025).

Since the pleural fluid was exudative in nature, the patient was tested for possible UTI but his urine analysis showed no bacteria; urine culture was negative. Hence, he was monitored off antibiotics. The nephrostomy tube placement was expected to improve the pleural effusion, but unfortunately, it continued to recur as shown on repeat CT scan in Figure 15.

**Figure 16:**
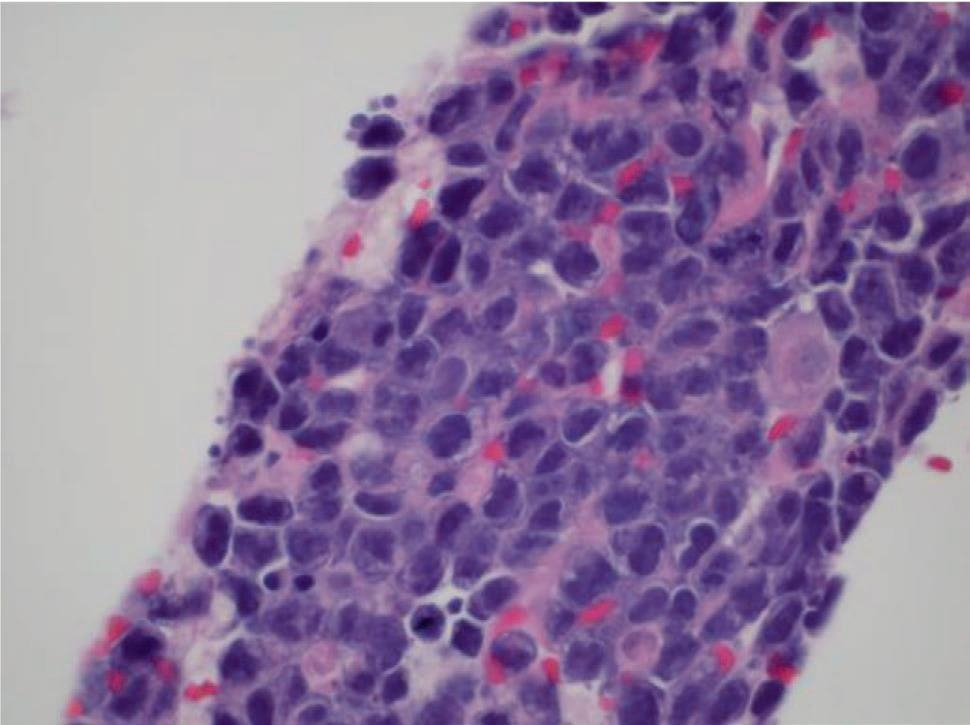
MT1 H and E stain.

**Figure 17:**
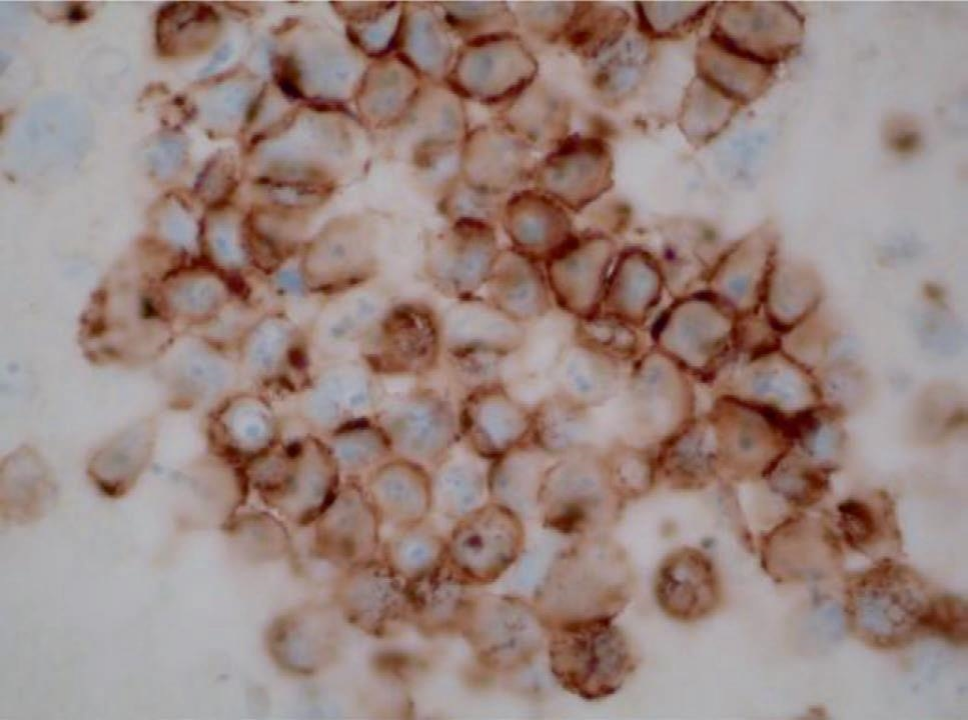
MT2 CD138 stain.

Meanwhile, pleural fluid cytology returned positive for pleomorphic myeloma as shown with the plasma cells on H and E stain in Figure 16 and confirmed by positive CD138 staining as in Figure 17. This finding was indicative of a MPE, which likely explained the exudative nature of the pleural fluid.

Further imaging with pelvic MRI revealed an ill‐defined, large lobular mass, suspected to be arising from the urinary bladder or prostate gland. Differential diagnoses included prostate cancer, extraosseous plasmacytoma, and extramedullary hematopoiesis. Cystoscopy was done that showed an inflamed bladder with old clots, but no active bleeding or visible tumors were identified. The patient then underwent a CT‐guided biopsy of the soft tissue pelvic mass, which confirmed the diagnosis of pleomorphic myeloma, with CD138 staining positive for myelomatous tissue. The appearance was identical to his previous bone marrow biopsy and pleural fluid findings.

The patient was started on palliative radiation therapy for symptomatic relief. The final diagnosis was extramedullary pleomorphic myeloma involving the pelvis, which caused bilateral ureteral obstruction, leading to bilateral hydronephrosis. This hydronephrosis, in turn, contributed to the occurrence of concurrent urinothorax, in addition to the MPE.

Given the aggressive nature of pleomorphic myeloma and its refractory response to systemic chemotherapy, the patient′s prognosis was extremely poor, with a limited life expectancy. After extensive discussions with the palliative care team, the decision was made to transition the patient to home hospice.

## 3. Discussion

MM is a condition that comprises < 10% of hematological malignancy and predominantly affects the elderly. It is characterized by the proliferation of malignant plasma cells and subsequent production of monoclonal protein/immunoglobulins [1]. Extramedullary disease is less common and usually predicts a poorer prognosis. One of these manifestations is pleural effusion which is uncommon and happens in approximately 6% of the patients [4].

MPE is a term that is used to describe collection of pleural fluid directly due to involvement of pleura with malignant plasma cells either because of direct pleural invasion, from extension of bony lesion or from a lung mass/plasmacytoma [1]. Most frequently, the causes of the effusion are infections due to associated hypogammaglobulinemia or bone marrow suppression caused by treatment, heart failure due to hyperviscosity or amyloidosis, renal failure, pulmonary embolism, and hypoalbuminemia. However, MPE is far less common and happens in less than 1% of MM patients [2]. The patients may show no respiratory symptoms or may have symptoms like dull chest pain, dyspnea, and dry cough, which are generally seen in pleural effusion. The diagnostic approach involves pleural fluid cytologic analysis and immunohistochemistry, with flow cytometry of pleural fluid being an excellent complementary diagnostic method to the traditional strategy. Pleural biopsy can also be done to confirm the results. Although IgG kappa is the most common type of MM, MPEs are mainly seen with the IgA subtype of myeloma cases ~80% of cases [1, 2]. Systemic chemotherapy aimed at MM, in conjunction with chest tube drainage or pleurodesis for palliation, is used for treatment purposes; however, prognosis remains poor with a median survival of 4 months [5].

On the other hand, urinothorax is defined as the presence of urine in the pleural space which is commonly seen with the finding of obstructive uropathy. Patients may present with similar symptoms of dyspnea, chest pain, abdominal pain, and decreased urine output as well. Two potential mechanisms of urinothorax have been identified including [1] obstructive uropathy that can result in extravasation of urine causing urinoma formation, which directly communicates through diaphragmatic pores into the pleural space or through a diaphragmatic defect and [2] indirectly via communication between retroperitoneal and pleural lymphatics. Pleural effusion usually develops when the rate of fluid accumulation exceeds the rate of fluid removal by the pleural lymphatics. For the diagnostic purpose, pleural fluid analysis will show pleural fluid to serum creatinine ratio of > 1.0 with either low or normal pH [2]. In our case, the pathophysiology of formation of urinothorax due to obstructive uropathy was more applicable given the findings.

While commonly a transudative effusion, an exudative pleural effusion does not exclude urinothorax but instead highlights its individuality [5]. In a literature review documented in 2017, 57 isolated cases of urinothorax from 1960 to 2016 were identified, out of which 28 cases (49%) were transudative, 13 cases (23%) were exudative, and 16 cases (28%) were not classified. In cases with alkaline range of pleural fluid pH > 7.40, it was found to be due to concomitant urinary tract infection causing urine alkalization [6, 7]. Moreover, prior systematic reviews have revealed negative pleural fluid cytology and only 9.3% (3/32 cases) of culture positive urinothorax in one study [4, 5].

## 4. Conclusion

This case highlights the complexity of diagnosing multifactorial pleural effusion, especially when laboratory findings overlap. Although MPE typically presents as an exudative effusion and urinothorax as a transudative one, there can be significant overlap. This needs a higher radar of suspicion for further differentials. Although elevated pleural fluid creatinine is a hallmark of urinothorax, renal impairment secondary to MM can complicate its interpretation. Our case is particularly unique because, despite meeting the criteria for urinothorax based on pleural fluid analysis—aside from the presence of exudative fluid—the patient′s effusion continued to worsen even after bilateral nephrostomy tube placement. The urine cultures were repeated, which showed no growth, hence ruling out possible urinary tract infection. This led to the thought of other possible differentials for having an exudative pleural effusion along with urinothorax. In the meantime, the cytology of pleural fluid and soft tissue biopsy of the pelvic mass resulted in the subsequent diagnosis of MPE, which was confirmed through positive CD138 staining on both with the appearance identical to each other and to prior bone marrow biopsy. We believe that no prior case of concomitant urinothorax and MPE has been documented in the literature before, given that the individual occurrence of both of these pathophysiologies themselves is very rare.

## Author Contributions

Dr. Prachi Bhanvadia, Dr. Amandeep Bawa, and Dr. Sara Samadani were involved in clinical management of the patient and case identification. Dr. Prachi Bhanvadia and Dr. Aarish Lalani drafted the manuscript and conducted the literature review. Dr. Laxman Wagle and Dr. Amandeep Bawa critically reviewed the manuscript for intellectual content and supervised the overall work.

## Funding

No funding was received for this manuscript.

## Consent

No written consent has been obtained from the patient as there is no patient identifiable data included in the case report.

## Conflicts of Interest

The authors declare no conflicts of interest.

## Data Availability

Data sharing is not applicable to this article as no datasets were generated or analyzed during the current study.
