# Correction to “A Rare Case of Concurrent Urinothorax and Myelomatous Pleural Effusion”

**DOI:** 10.1155/crpu/9861975

**Published:** 2026-07-05

P. Bhanvadia, A. Lalani, S. Samadani, A. Bawa, and L. Wagle. “A Rare Case of Concurrent Urinothorax and Myelomatous Pleural Effusion”, *Case Reports in Pulmonology* 2026 (2026): 6880861, 10.1155/crpu/6880861

In the article titled “A Rare Case of Concurrent Urinothorax and Myelomatous Pleural Effusion,” there was a spelling error in author Sara Samadani’s name in the author list, where Sara Samadami should have read Sara Samadani.

The online version of this article has been corrected accordingly.

We apologize for this error.

